# Prospects of Targeting the Gastrin Releasing Peptide Receptor and Somatostatin Receptor 2 for Nuclear Imaging and Therapy in Metastatic Breast Cancer

**DOI:** 10.1371/journal.pone.0170536

**Published:** 2017-01-20

**Authors:** Simone U. Dalm, Willemijne A. M. E. Schrijver, Anieta M. Sieuwerts, Maxime P. Look, Angelique C. J. Ziel - van der Made, Vanja de Weerd, John W. Martens, Paul J. van Diest, Marion de Jong, Carolien H. M. van Deurzen

**Affiliations:** 1 Department of Radiology & Nuclear Medicine, Erasmus MC, Rotterdam, The Netherlands; 2 Department of Pathology, University Medical Center Utrecht, Utrecht, The Netherlands; 3 Department of Medical Oncology and Cancer Genomics Netherlands, Erasmus MC Cancer Institute, Erasmus MC, Rotterdam, The Netherlands; 4 Department of Pathology, Erasmus MC, Rotterdam, The Netherlands; University of Alabama at Birmingham, UNITED STATES

## Abstract

**Background:**

The gastrin releasing peptide receptor (GRPR) and the somatostatin receptor 2 (SSTR2) are overexpressed on primary breast cancer (BC), making them ideal candidates for receptor-mediated nuclear imaging and therapy. The aim of this study was to determine whether these receptors are also suitable targets for metastatic BC.

**Methods:**

mRNA expression of human BC samples were studied by in vitro autoradiography and associated with radioligand binding. Next, *GRPR* and *SSTR2* mRNA levels of 60 paired primary BCs and metastases from different sites were measured by quantitative reverse transcriptase polymerase chain reaction. Receptor mRNA expression levels were associated with clinico-pathological factors and expression levels of primary tumors and corresponding metastases were compared.

**Results:**

Binding of GRPR and SSTR radioligands to tumor tissue correlated significantly with receptor mRNA expression. High *GRPR* and *SSTR2* mRNA levels were associated with estrogen receptor (ESR1)-positive tumors (p<0.001 for both receptors). There was no significant difference in *GRPR* mRNA expression of primary tumors versus paired metastases. Regarding *SSTR2* mRNA expression, there was also no significant difference in the majority of cases, apart from liver and ovarian metastases which showed a significantly lower expression compared to the corresponding primary tumors (p = 0.02 and p = 0.03, respectively).

**Conclusion:**

Targeting the GRPR and SSTR2 for nuclear imaging and/or treatment has the potential to improve BC care in primary as well as metastatic disease.

## Introduction

Breast cancer (BC) is the second most common cancer found in women and the fifth cause of cancer related death [1]. The disease is very heterogeneous. Different subtypes with distinctive morphological and molecular characteristics exist. The four major intrinsic BC subtypes are luminal A, luminal B, human epidermal growth factor 2 (HER2, *ERBB2*)-driven and basal-like BC [2, 3]. Treatment and prognosis of the disease are highly dependent on these subtypes; luminal A and luminal B tumors have a better prognosis than basal-like BC [2, 3]. Although multiple therapy options for BC exist, 20–30% of BC patients experience relapse with metastatic disease [4].

Peptide receptor mediated nuclear imaging and peptide receptor radionuclide therapy are methods successfully used in the clinic for imaging and treatment of neuroendocrine tumors [5]. These methods are based on targeting receptors that are overexpressed on cancer cells using radiolabeled peptide analogs. Regarding BC, multiple studies have demonstrated overexpression of the gastrin releasing peptide receptor (GRPR) and the somatostatin receptor 2 (SSTR2). In line with this, several pre-clinical as well as clinical studies demonstrated feasibility of imaging and/or treatment of BC with GRPR and SSTR2 radioligands with promising results, and indicated specific BC patients groups that can benefit from the application of these radioligands [6–10].

However, previous studies were solely based on primary BC while BC-related death is largely caused by metastatic disease. Targeting the GRPR and SSTR2 could thus especially be advantageous for treatment of metastatic BC.

In this study, we examined the *GRPR* and *SSTR2* mRNA expression levels of primary tumors and paired metastases, in order to evaluate whether nuclear imaging and therapy might also be beneficial for metastatic BC.

## Materials and Methods

### Human BC cases

Retrospectively, we selected 74 formalin-fixed paraffin-embedded (FFPE) primary BCs and 77 corresponding metastases from an existing database of the University Medical Center Utrecht and from the pathology archive of the Erasmus Medical Center [11, 12]. Fresh frozen (FF) tissue of 6 paired primary tumors and regional lymph node metastases were also included. Each specimen was reviewed by a pathologist (CHMvD) to confirm the presence of malignancy and to determine the percentage of tumor cells (cut-off point of >50% tumor cells). Inclusion criteria were: availability of clinico-pathological data, the presence of enough tumor tissue and good RNA quality to reliably determine RT-qPCR levels (see below). After applying these inclusion criteria, 68 primary tumors and 60 metastases remained, resulting in 60 paired primary BCs and metastases from different sites, including brain (n = 12), regional lymph nodes (n = 20), liver (n = 10), ovary (n = 5), lung (n = 5) and other sites (n = 8, consisting of bone (n = 2), uterus (n = 1), gastrointestinal tract (n = 2) and distant lymph node metastases (n = 3)). Clinico-pathological characteristics included age, primary tumor size, histological subtype, histological grade according to Bloom & Richardson [13], estrogen receptor (ER, *ESR1*) status, *ERBB2* status, and regional lymph node status.

Approval for the use of tissue samples was obtained from the Erasmus MC Medical Ethical Committee (MEC02·953) and adhered to the Code of Conduct of the Federation of Medical Scientific Societies in The Netherlands.

### RNA isolation and quantitative reverse transcriptase polymerase chain reaction (RT-qPCR)

Ten 10 μm slides were cut from the FFPE and 10×20 μm from the FF primary BCs and paired metastases. The first and last sections (5 μm) were stained with hematoxylin and eosin to guide macro-dissection of the tumor cells for RNA extraction. Total RNA was isolated from the macro-dissected FFPE sections with the AllPrep DNA/RNA FFPE Kit (Qiagen) and from the FF sections with RNA-B (Campro Scientific) according the manufacturer’s instructions. Nucleic acid concentrations were measured with a Nanodrop 1000 system. cDNA was generated for 30 min at 48°C with RevertAid H minus (ThermoFisher Scientific) and gene-specific pre-amplified with Taqman PreAmp Master mix (ThermoFisher Scientific) for 15 cycles, followed by Taqman probe—based real time PCRs according the manufacturer’s instructions in a MX3000P Real-Time PCR System (Agilent). The following gene expression assays were evaluated (all from ThermoFisher Scientific): *GRPR*, Hs01055872 m1; *SSTR2*, Hs0099356 m1; *ESR1*, Hs00174860_m1; *ERBB2*, Hs01001580_m1, and quantified relative to the average expression of *GUSB*, Hs9999908_m1; *HMBS*, Hs00609297_m1 and *TBP*, Hs00427620_m1 using the delta Cq method (dCq = 2ˆ(average Cq reference genes—Cq target gene)). Samples that resulted in amplifiable products within 25 cycles for this reference gene set at an input of 50 ng total RNA (91.2% of the samples) were considered to be of good quality to reliably determine RT-qPCR levels. Additional quality and quantity control measurements that were taken to ensure reliable RT-qPCR data analysis are described in the Supplemental Methods in S1 File.

In this study, we used *ESR1* and *ERBB2* mRNA expression levels to determine *ESR1* and *ERBB2* status (using a cut-off dCq for *ESR1*>1 and *ERBB2*>3.5 by optimal binning for n = 92 and n = 87 overlapping samples, respectively (See S1 File).

### Radioligands and in vitro autoradiography

The radiolabeled GRPR antagonist, JMV4168 [14], and the radiolabeled SSTR2 agonist, DOTA-Tyr^3^-octreotate (Mallinckrodt) were radiolabeled with ^111^In (Covidien) using quenchers to prevent radiolysis as previously described [15, 16]. Specific activity was 80 MBq/nmol for both radiotracers. Radiochemical purity and radiometal incorporation, measured by instant thin-layer chromatography on silica gel and high-pressure liquid chromatography as previously described, were >90% [16].

Slides (10 μM) of FF primary BC and paired metastases (n = 6 each) were used for autoradiography experiments. Tissue sections were incubated with 100 μL incubation buffer (167 mM Tris-HCL, 5 mM MgCl2, 1% BSA) containing 10^−9^ M of the radiolabeled peptide for 1 h, with and without 10^−6^ M unlabeled tracer to determine specificity of binding. Results of the autoradiography experiments were quantified using Optiquant (Perkin Elmer) and the percentage added dose (%AD) of the radioligand bound to the tumor tissue was used as an indirect measurement for the level of protein expression. Radioligand binding to primary tumors and paired metastasis was compared and correlated with the measured *GRPR* and *SSTR2* mRNA expression levels in corresponding FF tumor material. Furthermore, mRNA receptor expression measured in FF tumor material was correlated with mRNA receptor expression measured from FFPE tumor material of the same tumor. These correlation analyses were performed to demonstrate that mRNA expression of FFPE material could be used as a surrogate for radiotracer binding. The autoradiography experiments and quantification of the results were performed as described in the Supplemental Methods in S1 File.

### Statistics

For the analysis, the STATA statistical package v14.1 and SPSS version 23 were used. Variables were checked for normality prior to analysis. To compare mean values between two or more groups, the Student t-test or analysis of variance ANOVA were used. To compare values for primary and metastatic disease the paired t-test was applied. Pearson and Spearman correlations were calculated when appropriate. P≤0.05 were considered statistically significant.

## Results

### In vitro autoradiography

**Fig 1:**
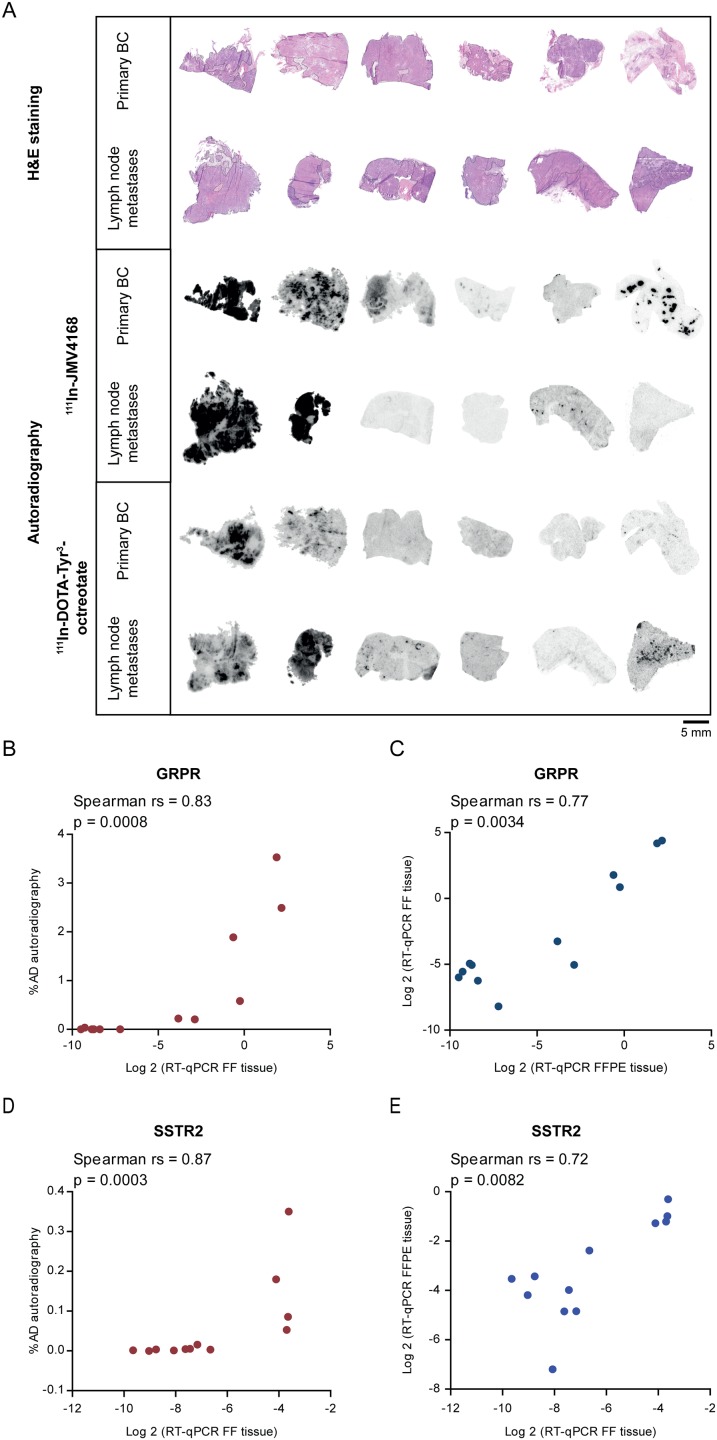
In vitro autoradiography of primary BC and corresponding regional lymph node metastases. A. Hematoxylin and eosin (H&E) staining and autoradiography results after incubating cells with the GRPR radioligand, ^111^In-JMV4168, and the SSTR2 radioligand, ^111^In-DOTA-Tyr^3^-octreotate. B+D. Correlation of quantified autoradiography results (% AD) with mRNA expression of fresh frozen (FF) tissue. C+E. Correlation of mRNA expression of FF and formalin fixed paraffin embedded (FFPE) tissue of the same tumor.

Six pairs of primary BCs and regional lymph node metastases (n = 12 samples) with varying mRNA receptor expression were analyzed for their ability to bind the GRPR radioligand, ^111^In-JMV4168, and the SSTR2 radioligand, ^111^In-DOTA-Tyr^3^-octreotate, using in vitro autoradiography. Fig 1A shows the in vitro autoradiography results of the paired samples. From the six paired samples analyzed, two cases showed specific binding of the GRPR and SSTR2 radioligands in both the primary tumor and the lymph node metastases. In two cases there was no binding of the GRPR radioligand and in three cases there was no binding of the SSTR2 radioligand in both the primary tumors and the lymph node metastases. In two cases binding of the GRPR radioligand was observed in the primary tumor but not in the lymph node metastasis, while in one case binding of the SSTR2 radioligand was observed in the lymph node metastasis, but not in the primary tumor.

When the %AD of the radiotracer bound to the FF tumor tissue was correlated with the mRNA receptor expression of the FF tumor material, a significant positive correlation was found for both GRPR (Spearman rs = 0.83, p = 0.0008) and SSTR2 (Spearman rs = 0.87, p = 0.0003) (Fig 1B+1D). Furthermore, correlation analysis of mRNA receptor expression levels quantified in FF and FFPE material of the same tumor, resulted in a significant positive correlation for both *GRPR* (Spearman rs = 0.77, p = 0.0034) and *SSTR2* (Spearman rs = 0.72, p = 0.0082) (Fig 1C+1E).

### Association of *GRPR* and *SSTR2* mRNA expression with clinico-pathological factors

Table 1 and S2 File show the patient characteristics, including the association of *GRPR* and *SSTR2* mRNA expression of primary BCs with clinico-pathological factors. High *GRPR* mRNA expression levels were significantly associated with low histologic grade, lobular subtype, *ESR1*-positive and *ERBB2*-negative tumors. High *SSTR2* mRNA expression levels were also significantly associated with lobular subtype and *ESR1*-positive tumors.

**Table 1:** Association of *GRPR* and *SSTR2* mRNA expression with clinico-pathological factors of primary BC ^a^.

Characteristic	No of patients	Percentage of patients	*GRPR* mRNA log2		*SSTR2* mRNA log2	
			Mean	SD	Mean	SD
**All patients in this cohort**	68	100	-2.90	3.8	-2.51	2.15
**Age at surgery (years)** ^b^						
≤ 40	11	16	-1.63	4.02	-2.23	2.13
41–55	27	39	-1.96	3.75	-2.08	2.38
56–70	22	32	-4.96	2.85	-3.07	1.94
> 70	7	10	-2.72	4.32	-2.90	2.04
*P*				*0*.*08*		*0*.*22*
**Tumor size** ^c^						
≤ 2 cm	25	36	-2.62	3.97	-2.61	2.24
2 ≤ 5 cm	29	42	-3.47	3.44	-2.32	2.16
> 5 cm	10	14	-2.00	4.22	-2.95	2.09
*P*				*0*.*51*		*0*.*72*
**Histopathological subtype** ^d^						
Ductal	55	80	-3.34	3.76	-2.82	2.15
Lobular	11	16	-0.80	3.44	-1.04	1.74
Other	2	3	-2.38	3.72	-2.04	1.26
*P*				***0*.*04***		***0*.*01***
**Bloom & Richardson grade** ^e^						
I + II	15	22	-1.23	3.57	-1.80	2.20
III	44	64	-3.66	3.91	-2.88	2.18
*P*				***0*.*04***		*0*.*12*
***ESR1* status** ^e^						
Negative	25	36	-6.52	1.68	-3.83	2.09
Positive	42	61	-0.79	3.04	-1.79	1.80
*P*				***<0*.*001***		***<0*.*001***
***ERBB2* status** ^e^						
Negative	46	67	-2.12	3.60	-2.44	2.26
Positive	11	16	-5.04	2.62	-2.7	2.23
*P*				***0*.*006***		*0*.*73*
**Regional lymph node status** ^e^						
Negative	15	22	-4.40	3.06	-2.96	2.33
Positive	44	64	-2.67	4.02	-2.48	2.01
*p*				*0*.*13*		*0*.*44*

^a^ Due to missing values numbers don’t always add up to 68

^b^ Receptor expression of ductal BC and lobular BC was compared using the student t-test.

^c^ P for Pearson correlation.

^d^ P for variance of ANOVA.

^e^ P for student t-test.

*GRPR* and *SSTR2* mRNA expression levels of the metastases were correlated with *ESR1* and *ERBB2* expression of the metastasis itself (Table 2). Similar to the primary tumors, high *GRPR* and *SSTR2* mRNA levels were significantly associated with *ESR1*-positive metastases. Furthermore, high *GRPR* mRNA status was significantly associated with *ERBB2*-negative metastases. Unlike the primary tumors, high *SSTR* mRNA levels were significantly associated with *ERBB2*-negative metastatic lesions.

**Table 2:** Association of receptor mRNA expression with *ESR1* and *ERBB2* status in BC metastases.

	*ER status* ^a^					*ERBB2 status* ^a^				
	Negative		Positive		*p*	Negative		Positive		*p*
	Mean	SD	Mean	SD		Mean	SD	Mean	SD	
**All metastases**										
No of patients	24		35			48		11		
*GRPR*	-6.26	2.76	-1.87	3.90	***<0*.*001***	*-2.67*	3.77	-7.98	2.15	***<0*.*001***
*SSTR2*	-3.95	1.64	-2.89	2.23	***0*.*04***	*-2.91*	1.96	-5.11	1.49	***<0*.*001***
**Regional lymph node metastases**										
No of patients	4		16			16		4		
*GRPR*	-7.0	1.96	-1.70	3.53	***0*.*003***	-1.70	3.54	-7.02	1.84	***0*.*002***
*SSTR2*	-4.03	0.97	-2.92	2.67	*0*.*20*	-2.77	2.47	-4.62	1.92	*0*.*16*
**All distant metastases** ^b^										
No of patients	20		19			32		7		
*GRPR*	-6.12	2.92	-2.01	4.27	***0*.*001***	-3.15	3.84	-8.52	2.26	***<0*.*001***
*SSTR2*	-3.94	1.76	-2.86	1.85	*0*.*07*	-2.98	1.69	-5.40	1.26	***0*.*001***

^a^ P for student t-test.

^b^ Numbers do not add up to 60 because for 1 patient ESR1 and ERBB2 were unknown.

### *GRPR* and *SSTR2* mRNA expression of primary BC vs. corresponding metastases

**Fig 2:**
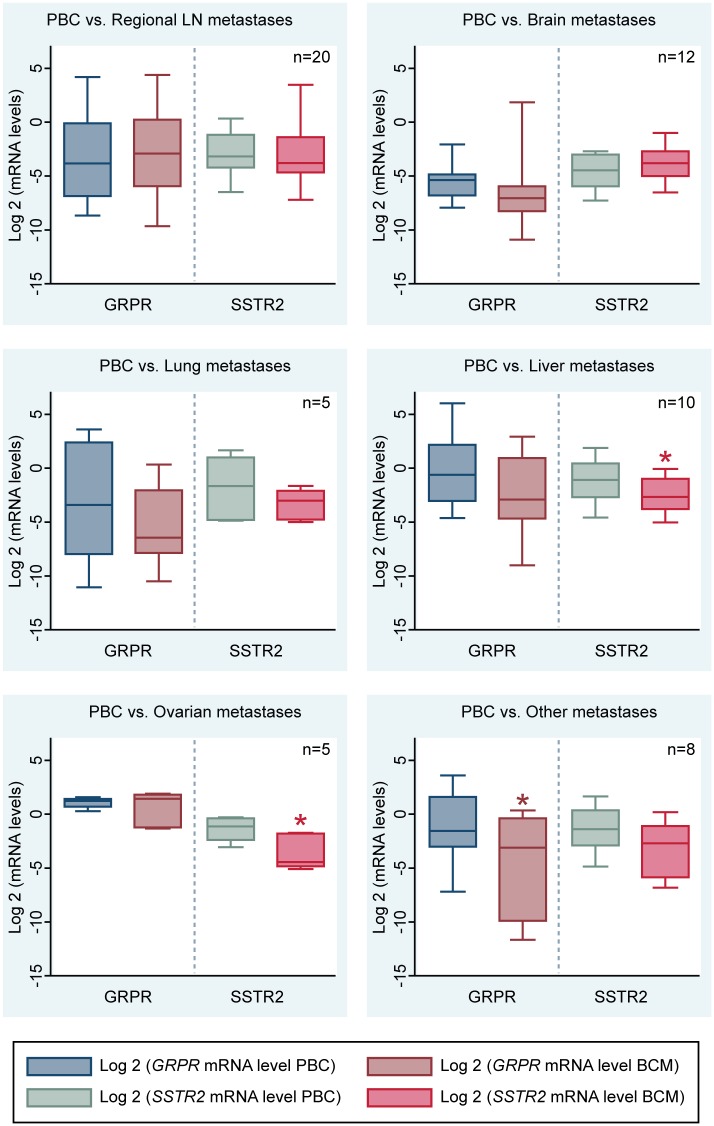
GRPR and SSTR2 mRNA levels in primary BC (PBC) and corresponding metastases (BCM). Significant differences are indicated by *.

Fig 2 shows the box plots of *GRPR* and *SSTR2* mRNA expression in primary tumors and corresponding metastases.

Comparison of receptor mRNA expression levels of primary tumors and corresponding metastases showed no significant difference in *GRPR* mRNA levels between primary tumors and corresponding regional lymph node and distant metastases in the brain, lung, liver and ovaries. However, in the group of metastases from other sites, *GRPR* mRNA expression levels were significantly lower in the metastases compared to the corresponding primary BC (p = 0.02).

Regarding *SSTR2* mRNA levels, there were no significant differences in *SSTR2* mRNA expression of the primary tumor and the paired metastasis in regional lymph nodes, brain, lung and other locations. However, *SSTR2* mRNA levels of liver and ovarian metastases were significantly lower compared to the expression in the corresponding primary BC (p = 0.02 and p = 0.03, respectively).

Next, we compared the receptor mRNA expression levels between distant metastases from various metastatic sites amongst each other. *GRPR* mRNA levels were significantly higher in the ovarian metastases (p = 0.03), while there were no significant differences in *SSTR2* mRNA expression levels in distant metastases from different sites.

In some cases studied (n = 11), there was a discordance regarding *ESR1* status of primary BCs and corresponding metastases. When studying the effect of change in *ESR1* status on receptor mRNA expression in primary tumors and paired metastasis, *GRPR* and *SSTR2* mRNA expression changed accordingly (higher *GRPR/SSTR2* mRNA expression in *ESR1*-positive lesions compared to *ESR1*-negative lesions) in the majority of the tumors. However, this difference was only significant (p<0.05) for *ESR1*-positive primary BCs with corresponding *ESR1*-negative metastases (n = 6). Discordance regarding *ERBB2* status was seen in 6 paired samples. In these samples a change in *ERBB2* status of primary BCs and corresponding metastases did not have a consistent effect on GRPR mRNA expression levels.

## Discussion

Targeting of GRPR and SSTR2 overexpressed on BC cells with radioligands can offer novel imaging and therapy options for BC. Previous clinical and preclinical studies reported promising results. However, these studies were restricted to primary BC, while metastases are the main cause of BC-related death. In this study, we compared *GRPR* and *SSTR2* mRNA expression levels in a unique dataset of primary BC and corresponding metastases to determine whether receptor-based imaging and/or therapy could also be useful for metastatic BC. For this purpose, we selected FFPE material of primary BCs and corresponding metastases from different sites, and compared mRNA receptor expression levels of the paired samples. Prior to this, we evaluated whether mRNA expression levels of tumor tissue properly represent radioligand binding, by correlating in vitro autoradiography results with mRNA expression levels of selected primary tumors and corresponding metastases with varying mRNA receptor expression. In line with previously published findings [10], we demonstrated that there was a high correlation between mRNA expression of the receptors and radiotracer binding, which suggests that mRNA expression levels can be used as a surrogate for radiotracer binding.

Next, we determined the *GRPR* and *SSTR2* mRNA expression of the paired primary BCs and metastases. When we associated receptor mRNA expression levels of primary BCs and metastases with clinico-pathological factors, we observed a significantly higher *GRPR* and *SSTR2* expression in both *ESR1*-positive primary BC and metastases. These findings are in agreement with our previous findings [10] and findings by Kumar et. al. [17] and Stoykow et al. [7]. The latter publication describes a clinical study in which the GRPR radioligand, ^68^Ga-RM2 was successfully used for imaging of BC lesions and imaging success-rate associated positively with ER and PR status. Furthermore, Prignon et al. [18] demonstrated that ^68^Ga-AMBA, a GRPR agonist, was better suited for monitoring response to hormonal treatment than ^18^F-FDG PET in an ER-positive BC model. In another study, van den Bossche et al.[19] published data indicating an estrogen-dependent regulation of SSTR expression in BC cell lines. Since ER-positive BC accounts for approximately 75% of the BC population, applying receptor targeted nuclear imaging and/or therapy using GRPR or SSTR2 radioligands could be beneficial for the majority of the BC population [2].

In paired primary tumors and metastases a change in *ESR1* expression from positive to negative resulted in a significant decrease in *GRPR* and *SSTR2* mRNA levels. This may indicate an *ESR1* dependent expression of *GRPR* and *SSTR2*, which is consistent with literature [19, 20]. Difference in *ERBB2* status in primary tumors and paired metastasis did not show a clear effect on *GRPR* mRNA expression, although these numbers were too small for reliable conclusions.

Comparison of *GRPR* and *SSTR2* mRNA levels of primary tumors and corresponding metastases resulted in similar *GRPR* mRNA expression in primary tumors and paired regional lymph nodes and distant metastases of the brain, lung, liver and ovaries. However, *GRPR* mRNA expression was significantly higher in primary tumors compared to corresponding metastases from other sites. Since this group is very diverse, containing metastases from distant lymph nodes, bone, uterus and metastases from the gastrointestinal tract, it is not possible to draw solid conclusions. Regarding *SSTR2*, mRNA expression levels were significantly lower in liver and ovarian metastases compared to the paired primary BC.

Combining our findings, both GRPR and SSTR2 are promising targets for nuclear imaging and/or therapy in primary and metastatic ER-positive BC, but GRPR seems more suitable due to its retained expression in the metastases. This finding is also supported by a previous study by our group, in which we demonstrated GRPR expression in 48/50 BCs [6], while SSTR2 was only expressed in 26/53 BCs (S.U. Dalm, C.H.M. van Deurzen, M. Melis, J.W. Martens and M. de Jong, unpublished data, 2014).

Since a substantial portion of BC patients experience relapse with metastatic disease, it is important to develop new treatment options for this late stage of disease. We showed that receptor mRNA expression levels were similar in primary tumors and corresponding metastases in the majority of the cases, implying that targeting these receptors for disease monitoring or therapy might improve BC patient care.

Biopsy material or excised tumors can be used to determine receptor expression by immunohistochemistry, RNA in situ, in vitro autoradiography or RT-qPCR [21]. Disease monitoring of receptor-positive tumors can then be performed by single photon emission computed tomography/computer tomography (SPECT/CT), positron emission tomography (PET)/CT or PET/magnetic resonance imaging using radioligands targeting these receptors. Also, dedicated breast PET cameras can be used. These dedicated cameras have improved sensitivity and specificity compared to whole body PET, because of a restricted field of view, resulting in higher cancer detection [22]. Furthermore, tumors can be treated with therapeutic radioligands. Another option is to use GRPR or SSTR2 radioligands for visualization of sentinel node metastases or as a guide for BC surgery (e.g. preoperative imaging, radioguided surgery) in patients with receptor positive primary tumors [23, 24].

The next step would be to perform clinical studies to investigate the feasibility of imaging primary tumors and metastases with radioligands targeting these receptors. One important aspect is to study physiological uptake of the radioligands in other organs, since this is of great importance for successful nuclear imaging and treatment. However, previous studies using radioligands targeting these receptors on other tumor types did not report on any alarming physiological uptake [5, 7].

Another potential interesting target for receptor mediated nuclear imaging and/or therapy is chemokine c-x-c motif receptor 4 (CXCR4), since high CXCR4 expression was reported in BC with high metastatic potential. Furthermore, radiotracers targeting the CXCR4 are available and have been tested successfully pre-clinically and clinically. Unfortunately, the CXCR4-targeted radiotracer available to us (Pentixafor) showed reduced affinity when labeled with ^111^In, hampering in vitro autoradiography experiments. We could therefore only analyze *CXCR4* mRNA expression levels as described in S3 File.

## Conclusion

The presented data indicates that nuclear based imaging and therapy has the potential to improve BC patient care in primary as well as in metastatic disease, by targeting GRPR and SSTR2. Both GRPR and SSTR2 radioligands, but especially GRPR radioligands, are promising for imaging and treatment of ER-positive primary and metastatic BC.

## Supporting Information

S1 File Supplemental methods. (DOCX)

S2 File Supplemental table. (DOCX)

S3 File Chemokine C-X-C motif receptor 4 expression in primary and metastatic breast cancer. (DOCX)
