# Supplementary material for: Prospects of Targeting the Gastrin Releasing Peptide Receptor and Somatostatin Receptor 2 for Nuclear Imaging and Therapy in Metastatic Breast Cancer
Source: PLoS One. 2017 Jan 20;12(1):e0170536. doi: 10.1371/journal.pone.0170536 (PMC5249060; doi:10.1371/journal.pone.0170536)
Supplement: S1 File — (DOCX) [file pone.0170536.s001.docx]

**Supplemental S1 file: Supplemental Methods**

**Quality and quantity control measurements for reliable quantitative reverse transcriptase polymerase chain reaction (RT-qPCR)**

For reliable RT-qPCR measurements, only samples that resulted in amplifiable products within 25 cycles for the used reference gene set at an input of 50 ng total RNA (91.2% of the samples) were considered to be of good quality for reliable determination of RT-qPCR levels. Furthermore, a serially diluted fresh frozen (FF) and formalin fixed paraffin embedded (FFPE) breast tumor sample was included in each experiment to evaluate the linear amplification and efficiencies for all genes included in the panel, and absence of amplification in the absence of reverse transcriptase. All gene transcripts were 100% efficient amplified (range 89%-113%) and were negative in the absence of reverse transcriptase. To ensure unbiased results from FF and FFPE samples, these 2 data sets were normalized based on the expression levels measured in a set of n=13 matched FF-FFPE samples.

**Estrogen receptor (ER/ESR1) and receptor tyrosine-protein kinase erbB-2 status/human epidermal growth factor 2 (ERBB2/HER2) status of the investigated samples**

Because data regarding ER and HER2 protein expression of our data set was incomplete, *ESR1* and *ERBB2* mRNA expression was used to determine *ESR1* and *ERBB2* mRNA status (using a dCq cut-off for *ESR1*>1 and *ERBB2*>3.5 by optimal binning for n=92 and n=87 overlapping samples, respectively (S1 Fig)). Because ER and HER2 are determined on protein level in daily clinical practice (using a scoring system according to national and international guidelines [1, 2]), we investigated whether the *ESR1* and *ERBB2* mRNA status accurately reflected the ER and HER2 protein status as reported in the pathology reports in samples with known receptor protein status. These cut-offs resulted for *ESR1* in a sensitivity of 0.88 and specificity of 0.85 and for *ERBB2* in a sensitivity of 0.89 and specificity of 0.97.

**
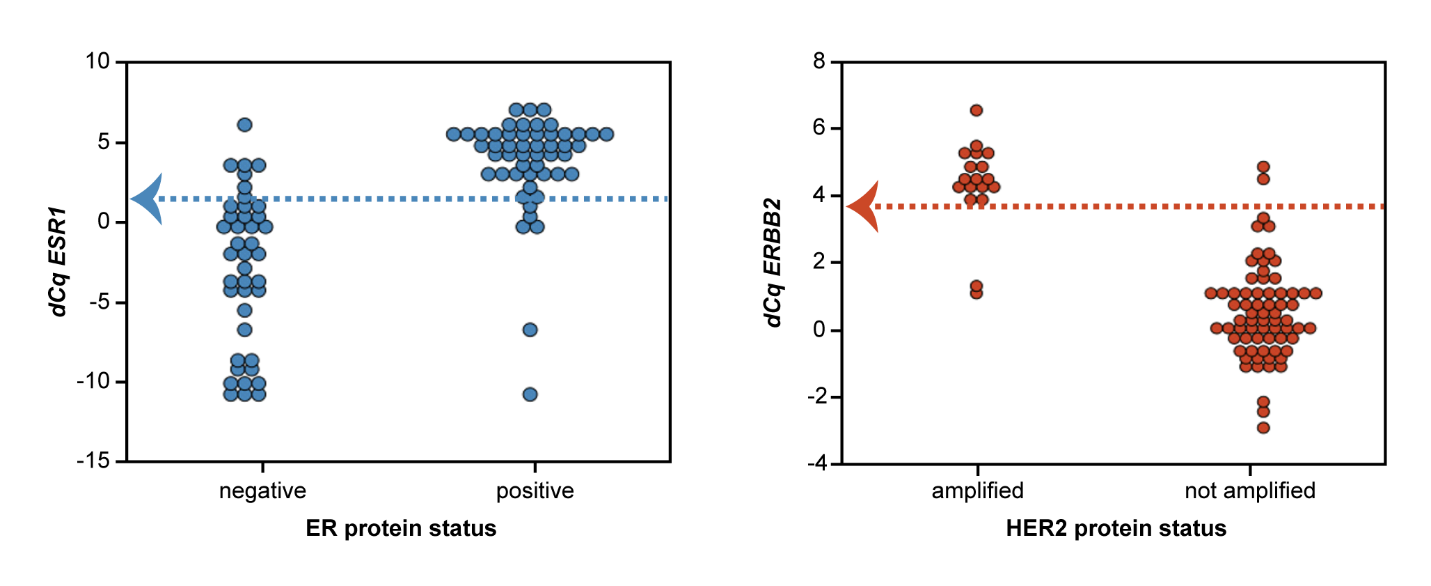
S1 Fig.** **Correlation of ER and HER2 protein status with ESR1 and ERBB2 mRNA levels.** Arrows indicate used cut-off value.

**In vitro autoradiography**

Fresh frozen tumor sections (10 μm) were incubated with 100 μL 10^-9^ M of the radioligands for 1 h, without and with 10^-6^ M unlabeled tracer. Octreotide (Covidien) and Tyr4-bombesin (Sigma-Aldrich) were used to block the somatostatin receptor and the gastrin releasing peptide receptor, respectively. Subsequently unbound radioligand was removed and slides were exposed to super-resolution phosphor screens (Perkin Elmer) for at least 24 h. Next, screens were read using the cyclone (Perkin Elmer) and the results were quantified using OptiQuant Software (Perkin Elmer). For this tumor containing regions, identified with the help of hematoxylin and eosin staining of adjacent tumor sections, were encircled and the digital light units/mm^2^ (DLU/mm^2^) were measured. Specific binding was determined by subtracting DLU/mm^2^ of blocked tissue sections from the DLU/mm^2^ of the unblocked sections (DLU/mm^2^_unblocked_ – DLU/mm^2^_blocked_ = DLU/mm^2^_specific_). Standards containing 1 μL drops of the radiotracer solution were also quantified and used to determine the percentage of added dose that was bound to the tumors (%AD = (DLU/mm^2^_specific_ / (DLU/mm^2^_standard_ × 100)) * 100%).

**References**

1. Breast Cancer Guideline, NABON 2012.

2. Wolff AC, Hammond ME, Hicks DG, Dowsett M, McShane LM, Allison KH, et al. Recommendations for human epidermal growth factor receptor 2 testing in breast cancer: American Society of Clinical Oncology/College of American Pathologists clinical practice guideline update. Arch Pathol Lab Med. 2014;138(2):241-56.
