# Supplementary material for: Prospects of Targeting the Gastrin Releasing Peptide Receptor and Somatostatin Receptor 2 for Nuclear Imaging and Therapy in Metastatic Breast Cancer
Source: PLoS One. 2017 Jan 20;12(1):e0170536. doi: 10.1371/journal.pone.0170536 (PMC5249060; doi:10.1371/journal.pone.0170536)
Supplement: S2 File — (DOCX) [file pone.0170536.s002.docx]

**Supplemental S2 file: Supplemental S1 Table**

**S1 Table. Overview of clinico-pathological characteristics of the primary BC associated with the site of the paired metastases ^a^**

|  |  |  | *Regional lymph node metastases n=20* | | *Brain metastases n=12* | | *Lung metastases n=5* | | *Liver metastases n=10* | | *Ovarian metastases n=5* | | *Other metastases n=8* | |
| --- | --- | --- | --- | --- | --- | --- | --- | --- | --- | --- | --- | --- | --- | --- |
| Characteristic | No of patients | Percentage of patients | No | % | No | % | No | % | No | % | No | % | No | % |
|  |  |  |  |  |  |  |  |  |  |  |  |  |  |  |
| **Age at surgery (years)** |  |  |  |  |  |  |  |  |  |  |  |  |  |  |
| ≤ 55 | 34 | 57 | 10 | 50 | 6 | 50 | 3 | 60 | 7 | 70 | 4 | 80 | 4 | 50 |
| ≥ 56 | 25 | 42 | 10 | 50 | 6 | 50 | 2 | 40 | 2 | 20 | 1 | 20 | 4 | 50 |
|  |  |  |  |  |  |  |  |  |  |  |  |  |  |  |
| **Tumor size** |  |  |  |  |  |  |  |  |  |  |  |  |  |  |
| < 2 - 5 cm | 48 | 80 | 18 | 90 | 9 | 75 | 3 | 60 | 8 | 80 | 2 | 40 | 8 | 100 |
| > 5 cm | 9 | 15 | 2 | 10 | 1 | 8 | 1 | 20 | 2 | 20 | 3 | 60 |  |  |
|  |  |  |  |  |  |  |  |  |  |  |  |  |  |  |
| **Histopathological subtype** |  |  |  |  |  |  |  |  |  |  |  |  |  |  |
| Ductal | 50 | 83 | 16 | 80 | 11 | 92 | 5 | 100 | 10 | 100 | 2 | 40 | 6 | 75 |
| Lobular | 9 | 15 | 3 | 15 | 1 | 8 |  |  |  |  | 3 | 60 | 2 | 25 |
| Other | 1 | 2 | 1 | 5 |  |  |  |  |  |  |  |  |  |  |
|  |  |  |  |  |  |  |  |  |  |  |  |  |  |  |
| **Bloom & Richardson grade** |  |  |  |  |  |  |  |  |  |  |  |  |  |  |
| I + II | 12 | 20 | 3 | 15 | 2 | 17 |  |  | 4 | 40 | 2 | 40 | 1 | 13 |
| III | 40 | 67 | 16 | 80 | 10 | 83 | 4 | 80 | 5 | 50 | 2 | 40 | 3 | 38 |
|  |  |  |  |  |  |  |  |  |  |  |  |  |  |  |
| ***ESR1* mRNA status** |  |  |  |  |  |  |  |  |  |  |  |  |  |  |
| Negative | 21 | 35 | 9 | 45 | 8 | 67 | 2 | 40 | 1 | 10 |  |  | 1 | 13 |
| Positive | 38 | 63 | 11 | 55 | 4 | 33 | 3 | 60 | 8 | 80 | 5 | 100 | 7 | 88 |
|  |  |  |  |  |  |  |  |  |  |  |  |  |  |  |
| **ERBB2 mRNA status** |  |  |  |  |  |  |  |  |  |  |  |  |  |  |
| Negative | 46 | 77 | 14 | 70 | 10 | 83 | 4 | 80 | 6 | 60 | 5 | 100 | 7 | 88 |
| Positive | 13 | 22 | 6 | 30 | 2 | 17 | 1 | 20 | 3 | 30 |  |  | 1 | 13 |

^a^ Due to missing values and rounding off numbers do not add up to 100%.
