# Supplementary material for: Prospects of Targeting the Gastrin Releasing Peptide Receptor and Somatostatin Receptor 2 for Nuclear Imaging and Therapy in Metastatic Breast Cancer
Source: PLoS One. 2017 Jan 20;12(1):e0170536. doi: 10.1371/journal.pone.0170536 (PMC5249060; doi:10.1371/journal.pone.0170536)
Supplement: S3 File — (DOCX) [file pone.0170536.s003.docx]

**Supplemental S3 file: Chemokine C-X-C motif receptor 4 expression in primary and metastatic breast cancer**

**Rationale**

Next to the somatostatin receptor 2 and the gastrin releasing peptide receptor, the chemokine c-x-c motif receptor 4 (CXCR4) is an interesting target for receptor mediated nuclear imaging and therapy of BC. Previous studies reported high CXCR4 expression in BC with high metastatic potential [1, 2]. Furthermore, CXCR4-targeting radiotracers have been synthesized and are successfully used preclinically and clinicaly [3-5]. We therefore also studied the *CXCR4* mRNA expression levels of the BC specimens (primary tumors and corresponding metastases) described in our manuscript. Unfortunately, the CXCR4 radiotracer available to us (pentaxifor) showed reduced receptor affinity when radiolabeled with ^111^In, hampering in vitro autoradiography experiments. Therefore, it was not possible to study whether CXCR4 radiotracer binding correlated with *CXCR4* mRNA expression, hampering interpretation of our data regarding CXCR4 as a target for receptor mediated nuclear imaging and/or therapy. However, in a previous study by Philipp-Abbrederis et al. [6] the authors reported on *CXCR4* mRNA expression in BC cell lines and CXCR4 targeted imaging of corresponding xenograft models, which suggests that *CXCR4* mRNA expression might be representable for radiotracer binding.

All experiments were conducted as described in the main manuscript text. To determine *CXCR4* mRNA expression the gene expression assay Hs01055872 m1 (ThermoFisher Scientific) was used.

**Results**

No association was found between the studied clinic-pathological factors and *CXCR4* mRNA expression of the primary BCs (S2 Table) and the metastases (S3 Table). Furthermore, there was no significant difference in *CXCR4* mRNA expression levels of primary tumors and corresponding regional lymph node and distant metastases (S2 Fig). When *CXCR4* mRNA expression of distant metastases from various sites was compared, *CXCR4* mRNA levels were significantly higher in liver metastases compared to metastases from other sites.

**S2 Table. Association of *CXCR4* mRNA expression with clinic-pathological factors of primary BC ^a^**

|  |  |  | *CXCR4* mRNA log2 | |
| --- | --- | --- | --- | --- |
| Characteristic | No of patients | Percentage of patients | Mean | SD |
| **All patients in this cohort** | 68 | 100 | 0.48 | 0.88 |
| **Age at surgery (years) ^b^** |  |  |  |  |
| ≤ 40 | 11 | 16 | 0.34 | 1.10 |
| 41-55 | 27 | 39 | 0.53 | 0.89 |
| 56-70 | 22 | 32 | 0.29 | 0.80 |
| > 70 | 7 | 10 | 1.09 | 0.67 |
| *P* |  |  |  | *0.32* |
| **Tumor size ^c^** |  |  |  |  |
| ≤ 2 cm | 25 | 36 | 0.34 | 0.88 |
| 2 ≤ 5 cm | 29 | 42 | 0.53 | 0.91 |
| > 5 cm | 10 | 14 | 0.53 | 0.89 |
| *P* |  |  |  | *0.70* |
| **Histopathological subtype ^d^** |  |  |  |  |
| Ductal | 55 | 80 | 0.50 | 0.90 |
| Lobular | 11 | 16 | 0.49 | 0.84 |
| Other | 2 | 3 | -0.32 | 0.14 |
| *P* |  |  |  | *0.96* |
| **Bloom & Richardson grade ^e^** |  |  |  |  |
| I + II | 15 | 22 | 0.18 | 0.86 |
| III | 44 | 64 | 0.62 | 0.89 |
| *P* |  |  |  | *0.10* |
| ***ESR1* status ^e^** |  |  |  |  |
| Negative | 25 | 36 | 0.54 | 0.90 |
| Positive | 42 | 61 | 0.41 | 0.87 |
| *P* |  |  |  | *0.58* |
| ***ERBB2* status ^e^** |  |  |  |  |
| Negative | 46 | 67 | 0.50 | 0.89 |
| Positive | 11 | 16 | 0.81 | 0.89 |
| *P* |  |  |  | *0.32* |
| **Regional lymph node status ^e^** |  |  |  |  |
| Negative | 15 | 22 | 0.66 | 1.01 |
| Positive | 44 | 64 | 0.40 | 0.82 |
| *p* |  |  |  | *0.33* |

^a^ Due to missing values numbers don’t always add up to 68.

^b^ Receptor expression of ductal BC and lobular BC was compared using the student t-test.

^c^ P for Pearson correlation.

^d^ P for variance of ANOVA.

^e^ P for student t-test

ESR1 = estrogen receptor, ERBB2 = receptor tyrosine-protein kinase erbB-2.

**S3 Table. Association of *CXCR4* mRNA expression with *ESR1* and *ERBB2* status in BC metastases**

|  | *ESR1 status ^a^* | | | | | *ERBB2 status ^a^* | | | | |
| --- | --- | --- | --- | --- | --- | --- | --- | --- | --- | --- |
|  | Negative | | Positive | | *p* | Negative | | Positive | | *p* |
|  | Mean | SD | Mean | SD |  | Mean | SD | Mean | SD |  |
| **All metastases** |  |  |  |  |  |  |  |  |  |  |
| No of patients | 24 | | 35 | |  | 48 | | 11 | |  |
| *CXCR4* | 0.27 | 1.28 | 0.29 | 1.07 | *0.95* | *0.17* | 1.14 | 0.74 | 1.13 | *0.15* |
| **Regional lymph node metastases** |  |  |  |  |  |  |  |  |  |  |
| No of patients | 4 | | 16 | |  | 16 | | 4 | |  |
| *CXCR4* | 0.04 | 0.91 | 0.14 | 0.90 | *0.85* | 0.10 | 0.88 | 0.21 | 1.00 | *0.86* |
| **All distant metastases ^b^** |  |  |  |  |  |  |  |  |  |  |
| No of patients | 20 | | 19 | |  | 32 | | 7 | |  |
| *CXCR4* | 0.31 | 1.36 | 0.41 | 1.20 | *0.82* | 0.21 | 1.26 | 1.05 | 1.14 | *0.12* |

^a^ P for student t test.

^b^ Numbers do not add up to 60 because for 1 patient *ESR1* and *ERBB2* were unknown.

ESR1 = estrogen receptor, ERBB2 = receptor tyrosine-protein kinase erbB-2.


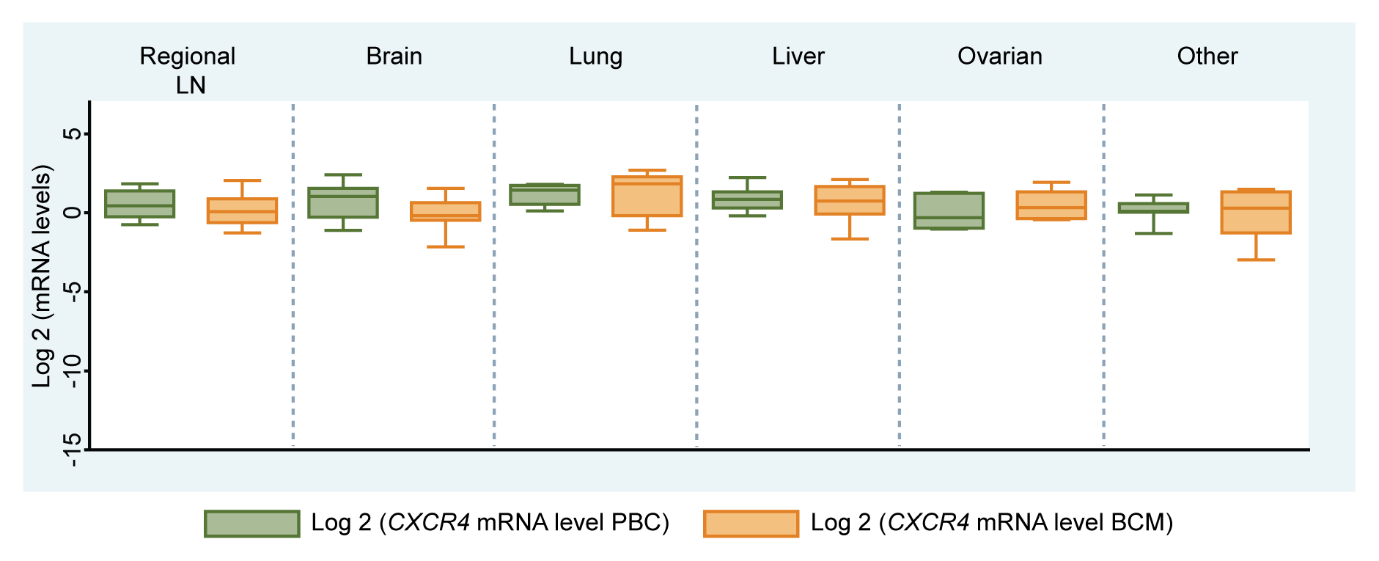


**S2 Fig. Comparison of *CXCR4* mRNA expression levels in primary BC and corresponding metastases. LN = lymph node, PBC = primary breast cancer and BCM = breast cancer metastases.**

**Discussion**

The current results do not show a significant association of high *CXCR4* mRNA expression with *ESR-1* negative tumors, which is in contrast to our previous study [7]. A potential explanation for this is the smaller sample size analyzed in this study (n=684 in our previous study vs. n=60 in this current study). Nevertheless, CXCR4 might be a good candidate for targeting of ER-negative BCs, that usually have a poor prognosis [8].

As is the case for SSTR2 and GRPR targeting radiotracers, CXCR4 mediated nuclear imaging of BC can potentially be used for disease monitoring, visualization of sentinel lymph node metastases or as a guide for BC surgery in patients with CXCR4 positive tumors. For clinical translation, an important aspect is physiological uptake of the radiotracer in other organs. To date, previous studies using the CXCR4 radiotracer, ^68^Ga-Pentixafor, in different tumor types did not report on any alarming results [5, 9]. In addition, peptide receptor radionuclide therapy using CXCR4 radiotracers coupled to therapeutic radionuclides can provide novel therapeutic options for CXCR4 positive BC patients.

However, a recent study by Vag et al. [9] reported on CXCR4-mediated nuclear imaging in BC patients with disappointing results. The authors performed ^68^Ga-Pentixafor imaging in 3 BC patients. Radiotracer uptake in the lesions was low or absent and when compared to ^18^F-FDG PET, ^18^F-FDG uptake was higher than that of ^68^Ga-Pentixafor. A possible explanation is the absence of (sufficient) CXCR4 mRNA expression on the cell membrane. However, the amount of BC patients included in this study is low.

To conclude, our study demonstrates that there is no significant difference in *CXCR4* mRNA expression levels in primary tumors and corresponding metastases. Larger studies are needed to correctly evaluate the value of targeting CXCR4 for nuclear imaging and/or therapy in BC patients.

**References**

1. Hung CS, Su HY, Liang HH, Lai CW, Chang YC, Ho YS, et al. High-level expression of CXCR4 in breast cancer is associated with early distant and bone metastases. Tumour Biol. 2014;35(2):1581-8.

2. Yang P, Liang SX, Huang WH, Zhang HW, Li XL, Xie LH, et al. Aberrant expression of CXCR4 significantly contributes to metastasis and predicts poor clinical outcome in breast cancer. Curr Mol Med. 2014;14(1):174-84.

3. Azad BB, Chatterjee S, Lesniak WG, Lisok A, Pullambhatla M, Bhujwalla ZM, et al. A fully human CXCR4 antibody demonstrates diagnostic utility and therapeutic efficacy in solid tumor xenografts. Oncotarget. 2016;17(11):12344-58.

4. Herhaus P, Habringer S, Philipp-Abbrederis K, Vag T, Gerngross C, Schottelius M, et al. Targeted positron emission tomography imaging of CXCR4 expression in patients with acute myeloid leukemia. Haematologica. 2016;101(8):932-40.

5. Lapa C, Luckerath K, Kleinlein I, Monoranu CM, Linsenmann T, Kessler AF, et al. (68)Ga-Pentixafor-PET/CT for Imaging of Chemokine Receptor 4 Expression in Glioblastoma. Theranostics. 2016;6(3):428-34.

6. Philipp-Abbrederis K, Herrmann K, Knop S, Schottelius M, Eiber M, Luckerath K, et al. In vivo molecular imaging of chemokine receptor CXCR4 expression in patients with advanced multiple myeloma. EMBO Mol Med. 2015;7(4):477-87.

7. Dalm SU, Sieuwerts AM, Look MP, Melis M, van Deurzen CH, Foekens JA, et al. Clinical Relevance of Targeting the Gastrin-Releasing Peptide Receptor, Somatostatin Receptor 2, or Chemokine C-X-C Motif Receptor 4 in Breast Cancer for Imaging and Therapy. Journal of nuclear medicine : official publication, Society of Nuclear Medicine. 2015;56(10):1487-93.

8. Yersal O, Barutca S. Biological subtypes of breast cancer: Prognostic and therapeutic implications. World J Clin Oncol. 2014;5(3):412-24.

9. Vag T, Gerngross C, Herhaus P, Eiber M, Philipp-Abbrederis K, Graner FP, et al. First Experience on Chemokine Receptor CXCR4 Targeted Positron Emission Tomography (PET) Imaging in Patients with Solid Cancers. J Nucl Med. 2016.
